# A preliminary study on drug switching strategy for second-line therapy after combination treatment of tyrosine kinase inhibitors and immune checkpoint inhibitors for unresectable hepatocellular carcinoma

**DOI:** 10.3389/fphar.2022.998534

**Published:** 2022-09-30

**Authors:** Renguo Guan, Chengyou Yu, Shaohua Li, Jie Mei, Wei Wei, Rongping Guo

**Affiliations:** ^1^ Department of Hepatobiliary Oncology, Sun Yat-Sen University Cancer Center, Guangzhou, China; ^2^ State Key Laboratory of Oncology in South China, Guangzhou, China; ^3^ Collaborative Innovation Center for Cancer Medicine, Guangzhou, China

**Keywords:** hepatocellular carcinoma, combination treatment, tyrosine kinase inhibitor, immune checkpoint inhibitor, drug switching, second-line therapy

## Abstract

**Background:** Combination treatment with tyrosine kinase inhibitors (TKIs) and immune checkpoint inhibitors (ICIs) has been widely used in patients with unresectable hepatocellular carcinoma (uHCC). As no standard guidelines exist for second-line therapy after failure of combination treatment, this study aimed to determine a better drug-switching strategy.

**Methods:** A total of 785 patients with uHCC who initially received a combination treatment of TKIs and ICIs between January 2017 and December 2021 at our center were screened. After applying the inclusion and exclusion criteria, a total of 102 patients were included in the study. Based on drug switching strategy, patients were divided into a single drug-switching group (A group, n = 49) and a double drug-switching group (B group, *n* = 53). The comparative effectiveness between groups A and B was assessed based on treatment response and survival time. Second progression-free survival (SPFS) and overall survival (OS) were compared using the Kaplan-Meier method and log-rank test.

**Results:** Compared to group B, group A had a higher overall response rate (16.3% vs. 3.8%; *p* = 0.0392) and disease control rate (61.2% vs. 49.1%; *p* = 0.238). The median SPFS in group A was longer than that in group B (5.47 vs. 3.8 months; HR = 1.70, *p* = 0.0176). In the second-line therapy, the inclusion of lenvatinib resulted in a better SPFS than other TKI treatments (5.53 vs. 2.83 months, *p* = 0.0038).

**Conclusion:** After the failure of the combination treatment of TKIs and ICIs, single-drug switching significantly prolonged median SPFS in uHCC patients, and retaining lenvatinib resulted in the survival benefit of single-drug switching.

## Introduction

Hepatocellular carcinoma (HCC) is one of the most frequent malignancies and the third leading cause of cancer deaths worldwide (Sung et al., 2021). Hepatectomy and liver transplantation are the most effective treatments for HCC. Because of its unobvious symptoms, many patients with HCC are diagnosed at advanced stages, and hence have a low survival rate (Yang and Heimbach, 2020). Systemic therapy, including systemic chemotherapy and local interventional therapy, is the predominant therapeutic modality for unresectable HCC (uHCC). A previous study has demonstrated that compared to the best supportive care, metronomic capecitabine was an alternative choice to sorafenib with better efficacy and safety (De Lorenzo et al., 2018). Local therapy also brings significant benefits to uHCC patients. Recently, a multi-center propensity score-matched analysis has confirmed that transarterial infusion chemotherapy with FOLFOX was an effective and safe therapy that improved the survival of advanced hepatocellular carcinoma (Li et al., 2021). Transarterial chemoembolization (TACE) is recommended as the standard therapy for HCC patients with BCLC stage B (Mei et al., 2021).

In recent years, with the progress in research and clinical application of targeted and immunotherapy drugs, the prognosis of patients with uHCC has significantly improved (Llovet et al., 2008; Kudo et al., 2018; Finn et al., 2020; Yau et al., 2022). However, less than 20% of patients with uHCC benefited from immune checkpoint inhibitors (ICIs) monotherapy (Rizzo et al., 2021). The role of ICI-based combinations warrants further evaluation, and it is exciting that better prognosis benefits were demonstrated with combination therapy of tyrosine kinase inhibitors (TKIs) and ICIs than with monotherapy (Cheng et al., 2020a). The IMbrave 150 study reported that atezolizumab plus bevacizumab could result in better overall survival (OS) and progression-free survival (PFS) in the Chinese subpopulation (Qin et al., 2021). Moreover, ^90^Yttrium transarterial radioembolization has an established synergism with atezolizumab plus bevacizumab treatment by enhancing antigen presentation and reducing the infiltration of immunosuppressive cells (Di Federico et al., 2022). The KEYNOTE 524 reported significant improvements with pembrolizumab plus lenvatinib, with an objective response rate (ORR) of 46% (Llovet et al., 2022). Camrelizumab combined with apatinib as the first-line therapy can significantly prolong PFS and OS in patients with advanced HCC when compared with sorafenib, and the independent data monitoring committee judged that the primary endpoint of the study met the protocol-preset superiority criteria (SHR-1210-III-310). Thus, combination treatment with TKIs and ICIs has been applied as a first-line treatment for patients in China (Zhao and Cai, 2021). Owing to tumor heterogeneity, tumor progression still occurs in patients with HCC receiving first-line treatment. Although there are some options for second-line treatment (Choi et al., 2020; Zhang et al., 2020), there is a lack of widely accepted guidelines for switching therapy.

To our knowledge, real-world outcomes of switching therapy and a comparison of its efficacy have not been reported. Based on real-world data from clinical practice, this study aimed to explore the effect of the mode of switching therapy on the prognosis of uHCC after first-line systemic therapy failure, and thus providing a reference for larger prospective clinical studies in the future to guide the complete treatment of HCC.

## Patients and methods

### Ethics statement

The Institutional Review Board of the Ethics Committee of Sun Yat-sen University Cancer Center approved this study (B2020-190-01). All procedures involving human participants were performed in accordance with the Declaration of Helsinki. Written informed consent was obtained from all patients for anonymized information published in this article.

### Study population

**FIGURE 1:**
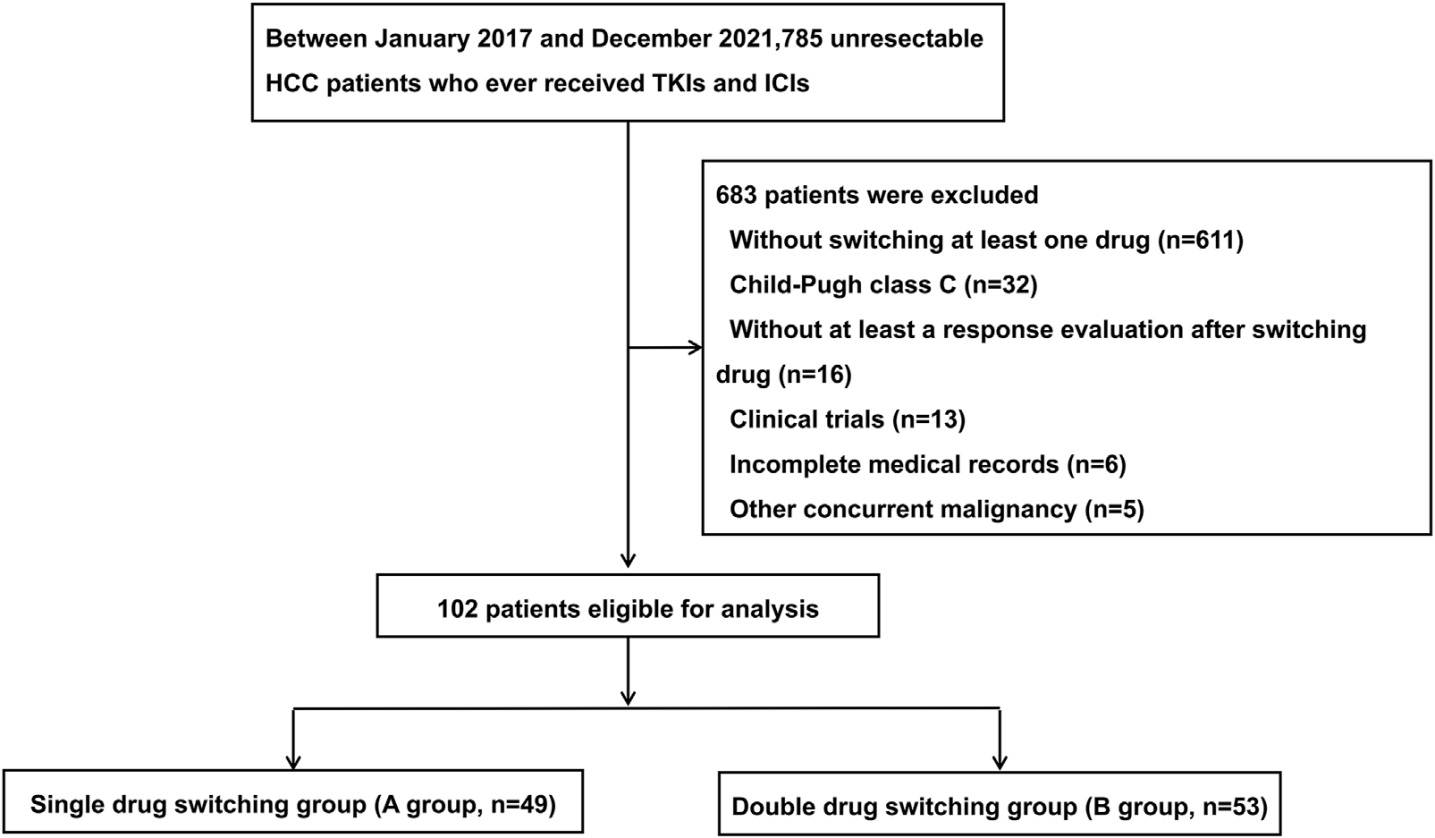
The flow chart of the disposition process of patients. HCC, hepatocellular carcinoma; TKIs, tyrosine kinase inhibitors (TKIs); ICIs, immune checkpoint inhibitors.

Patients with uHCC who received TKIs and ICIs at the Department of Liver Surgery of Sun Yat-sen University Cancer Center between January 2017 and December 2021 were included in this retrospective analysis. The inclusion criteria for patients were as follows: 1) aged 18–75 years; 2) diagnosed with uHCC according to the AASLD practice guidelines (Marrero et al., 2018); 3) Child-Pugh class A or B; 4) at least one measurable lesion based on the modified Response Evaluation Criteria in Solid Tumors (mRECIST) criteria (Llovet and Lencioni, 2020); and 5) switched to at least one systemic therapy drug after tumor progression. The exclusion criteria of the patients were as follows: 1) presence of other malignant tumors; 2) no response evaluation after switching therapy; 3) incomplete baseline and follow-up data; and 4) clinical trials participants. A total of 102 patients with HCC were included in this study based on the inclusion and exclusion criteria. The details of the initial combination treatment and second-line treatment for the 102 uHCC patients are listed in Tables 1 and 2, respectively. All the patients were classified into two groups: group A (*n* = 49) and group B (*n* = 53), based on the mode of switching therapy. The group A included uHCC patients who switched to one systemic therapy drug after tumor progression, while the group B included patients who switched to two systemic therapy drugs after tumor progression. A flowchart of the patient disposition process is shown in Figure 1.

**TABLE 1:** Initial combination treatment of the patients.

Tyrosine kinase inhibitors	Immune checkpoint inhibitors	A group (*n* = 49)	B group (*n* = 53)
	Camrelizumab	2	2
	Tislelizumab	0	1
	Toripalimab	0	9
Apatinib	Keytruda	1	0
	Nivolumab	0	1
	Sintilimab	0	1
	Camrelizumab	1	0
	Toripalimab	21	7
Lenvatinib	Keytruda	6	4
	Nivolumab	0	4
	Sintilimab	8	7
	Durvalumab	0	1
	Sintilimab	1	1
Regorafenib	Keytruda	0	3
	Durvalumab	1	0
	Toripalimab	4	6
Sorafenib	Nivolumab	2	0
	Sintilimab	1	7

uHCC: unresectable HCC.

**TABLE 2:** Second-line treatment given to the patients.

Tyrosine kinase inhibitors	Immune checkpoint inhibitors	A group (*n* = 49)	B group (*n* = 53)
Apatinib	Camrelizumab	0	7
	Toripalimab	3	0
	NA	0	1
Lenvatinib	Camrelizumab	5	3
	Toripalimab	6	1
	Keytruda	4	1
	Nivolumab	2	0
	Sintilimab	13	8
	Durvalumab	3	0
	Tislelizumab	6	3
	NA	0	8
Regorafenib	Camrelizumab	1	0
	Tislelizumab	0	1
	Durvalumab	0	1
	Sintilimab	2	0
	NA	0	5
Sorafenib	Camrelizumab	0	1
	Toripalimab	2	0
	Sintilimab	1	1
	NA	0	3
Bevacizumab	Atezolizumab	0	8
	Durvalumab	0	1
	Lenvatinib	1	0
	NA	0	0

Abbreviation: uHCC: unresectable HCC.

### Treatment procedure

Patients with uHCC received a combination of ICIs and TKIs as initial treatment. TKIs including regorafenib, apatinib, sorafenib, and lenvatinib were administered orally once daily. The ICIs included PD-1 and PD-L1 inhibitors which were intravenously injected every 3 weeks. The initial doses of TKIs and ICIs used are listed in Supplementary Table S1. The interval between the initiation of ICIs and TKIs was less than 7 days. Combination treatment with ICIs and TKIs was continued until the occurrence of disease progression or intolerable toxicity. After tumor progression, the decision to switch drugs was based on resistance to TKIs and ICIs or liver function. All patients with uHCC underwent scheduled enhanced computed tomography or magnetic resonance imaging assessment every 2-3 months.

### Data collection and clinical outcomes

All baseline data before second-line treatment were retrieved from medical records and imaging examinations, including age, sex, Child-Pugh class, α-fetoprotein (AFP), protein induced by vitamin K absence or antagonist-II (PIVKA-II), albumin, total bilirubin (TB), etiology, Barcelona Clinic Liver Cancer stage (BCLC stage), alanine aminotransferase (ALT), aspartate aminotransferase (AST), alkaline phosphatase (ALP), progressive, macroscopic portal vein invasion, portal hypertension, and extrahepatic metastases. Tumor response to treatment was defined as complete response (CR), partial response (PR), stable disease (SD), or progressive disease (PD), based on the mRECIST criteria.

Second progression-free survival (SPFS) and OS were the clinical outcomes of interest. SPFS was defined as the interval from the initiation of second-line treatment to tumor progression, while OS was measured from the initiation of second-line treatment to death. The secondary outcomes included objective response rate (ORR) and disease control rate (DCR). ORR was defined as achieving CR or PR, and DCR was defined as achieving CR, PR, or SD. Treatment-related adverse events (AEs) were evaluated using the National Cancer Institute Common Terminology Criteria for Adverse Events (CTCAE) version 4.0.

### Statistical analysis

The baseline characteristics were compared between the different modes of switching therapy. Continuous variables with normal distribution were expressed as means and standard deviations and those with abnormal distribution were expressed as medians and interquartile ranges. Continuous variables were analyzed using an unpaired Student’s t-test for parametric data and the Mann–Whitney rank sum test for non-parametric data. Categorical variables were compared using Pearson’s chi-squared test or Fisher’s exact test. The survival analysis between the different treatment groups was performed by plotting Kaplan-Meier curves and their differences were verified using the log-rank test. Univariate Cox regression analysis was used to identify survival-associated factors, which were sequentially subjected to multivariate Cox regression analysis to identify the independent prognostic factors. All statistical analyses were performed using the SPSS software (version 20.0), MedCalc (version 20.027), and R software (version 4.1.1). Two-sided *p*-values <0.05 were considered statistically significant.

## Results

### Patient characteristics

The clinical characteristics of the patients and therapy given are summarized in Table 3. The median age of the study population was 54 years old. The majority of the patients were Child-Pugh class A (*n* = 91, 89.22%) and chronically infected with the hepatitis B virus (*n* = 92, 90.2%). Of the patients, 89.2% received other treatments, such as radiofrequency ablation, radiotherapy, hepatic artery infusion chemotherapy, transhepatic arterial chemotherapy, and embolization. Males were predominant (*n* = 80, 78.43%) and 2/3rd of the patients were in BCLC stage C (*n* = 76, 74.51%). The patients with extrahepatic metastases were approximately 60%. Almost half of the patients had macroscopic portal vein invasion (*n* = 41, 40.2%) and single intrahepatic progression (*n* = 42, 41.18%). In addition, 36.27% of patients had portal hypertension. The duration of first-line treatment and baseline characteristics were not significantly different between the groups (*p* >0.05).

**TABLE 3:** Clinicopathological characteristics of hepatocellular carcinoma patients.

Characteristics	Total (*n* = 102)	A group (*n* = 49)	B group (*n* = 53)	*p*-value
Age, years	54 (43, 63)^a^	53.9 ± 12.5^b^	51 ± 12.9^b^	0.194
Male sex, n (%)	80 (78.43)	39 (79.59)	41 (77.36)	0.7841
Child-Pugh class, n (%)				0.1003
A	91 (89.22)	41 (83.67)	50 (94.34)	
B	11 (10.78)	9 (16.33)	3 (5.66)	
AFP, n (%)				0.2913
≥ 400 ng/ml	44 (43.1)	18 (36.7)	26 (49.1)	
<400 ng/ml	58 (56.9)	31 (63.3)	27 (50.9)	
PIVKA-II, n (%)				0.1049
≥1,000 mAU/mL	47 (46.1)	18 (36.7)	29 (54.7)	
<1,000 mAU/mL	55 (53.9)	31 (63.3)	24 (45.3)	
Albumin, median (IQR), g/dL	4 (3.8, 4.4)	4.1 (3.8, 4.4)	4.1 (3.9, 4.5)	0.671
Total bilirubin, median (IQR), mg/dL	13.3 (10, 19.7)	13.5 (10.7, 21.2)	13.1 (10, 17.8)	0.567
Etiology, n (%)				0.3848
Yes	92 (90.2)	46 (93.88)	46 (86.79)	
No	10 (9.8)	3 (6.12)	7 (13.21)	
BCLC stage, n (%)				0.7329
A	3 (2.94)	2 (4.08)	1 (1.89)	
B	23 (22.55)	10 (20.41)	13 (24.53)	
C	76 (74.51)	37 (75.51)	39 (73.58)	
Macroscopic portal vein invasion, n (%)	41 (40.2)	19 (38.78)	22 (41.51)	0.7784
ALT, median (IQR)	34.65 (24.4, 55.4)	28.8 (21.9, 52.5)	43.3 (29,65.3)	0.082
AST, median (IQR)	41.25 (30.9, 65.6)	39.2 (29.2, 63.9)	43.4 (33.3,75)	0.325
ALP, median (IQR)	107.9 (76.4,148)	100.5 (71.5, 138.9)	110.8 (82, 165.6)	0.190
Progressive-pattern				0.3926
Only extrahepatic progression	28 (27.45)	14 (28.57)	14 (26.415)	
Only intrahepatic progression	42 (41.18)	17 (34.69)	25 (47.17)	
Both	32 (31.37)	18 (36.73)	14 (26.415)	
Extrahepatic metastases	58 (56.86)	29 (59.18)	29 (54.72)	0.6491
Lymph node	34 (33.33)	16 (32.65)	18 (33.9)	
Lung	34 (33.33)	11 (22.45)	23 (43.4)	
Peritoneum	10 (9.8)	4 (8.16)	6 (11.3)	
Bone	9 (8.8)	5 (10.2)	4 (7.5)	
Others	12 (14.7)	4 (8.16)	8 (15)	
Portal hypertension	37 (36.27)	19 (38.78)	18 (33.96)	0.6135
Other treatments				0.8559
With	91 (89.2)	44 (89.8)	47 (88.7)	
Without	11 (10.8)	5 (10.2)	6 (11.3)	
Time interval of drug switching (days)	18 (9,28)	18 (12,25)	16 (7,29)	0.6294
Duration of first-line treatment (months)	6.5 (4.3,11.4)	8.2 (4.2,14.1)	5.6 (4.3,8.4)	0.132

a median (IQR).

b mean ± standard deviation.

### Treatment response

The treatment responses are summarized in Table 4. Based on mRESIST, four patients had CR, six patients had PR, forty-six patients had SD and forty-six patients had PD. ORR and DCR were 9.8% and 54.9%, respectively. Notably, the ORR was higher in group A (16.3%) than in group B (3.8%) (*p* = 0.0392). A higher DCR was observed in group A than in group B (61.2% vs. 49.1%; *p* = 0.238). Collectively, the single drug switching strategy might provide clinical benefits to patients with uHCC.

**TABLE 4:** Treatment response of patients.

Evaluation (mRECIST)	Total	A group	B group
Complete response	4	2	2
Partial response	6	6	0
Stable disease	46	22	24
Progressive disease	46	19	27
Objective response rate^#^ (%)	9.8	16.3	3.8
Disease control rate* (%)	54.9	61.2	49.1
Death	31	16	15

Abbreviation: mRECIST, modified response evaluation criteria in solid tumors.

# Two-sided *p*-value = 0.0392.

* Two-sided *p*-value = 0.238.

### Comparison of the effectiveness of the switching modes

**FIGURE 2:**
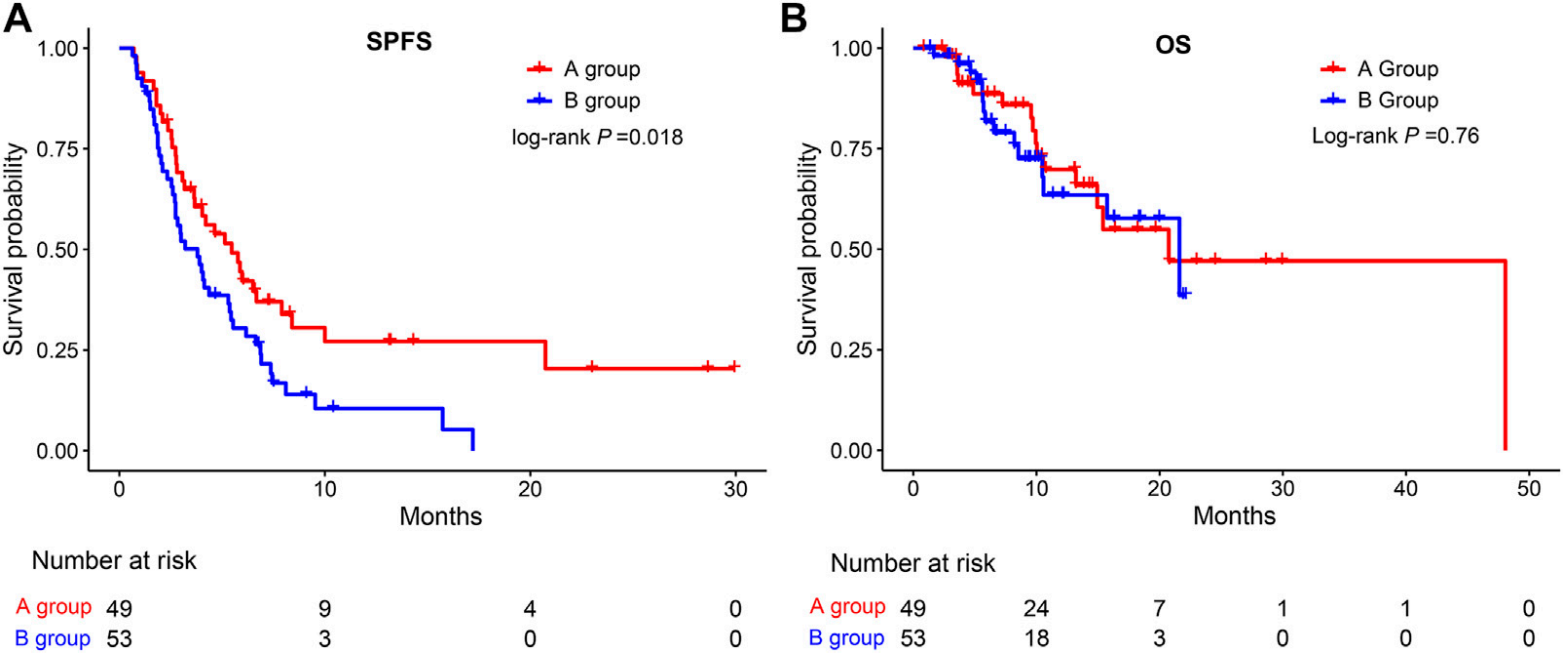
Kaplan-Meier survival curves for SPFS **(A)** and OS **(B)** of patients in the A group and the B group. SPFS, second progression-free survival; OS, overall survival.

As shown in Figures 2A,B, the median SPFS was significantly longer in group A (5.47 months) than in group B (3.8 months) (HR = 1.70, 95%CI: 1.089–2.641, *p* = 0.0176), while there was no significant difference in OS between group A and group B (HR = 1.12, 95%CI: 0.55–2.26, *p* = 0.7556). The median OS in groups A and B were 20.7 and 21.6 months, respectively.

### Single drug switching extended second progression-free survival of patients with BCLC Stage A or B

**FIGURE 3:**
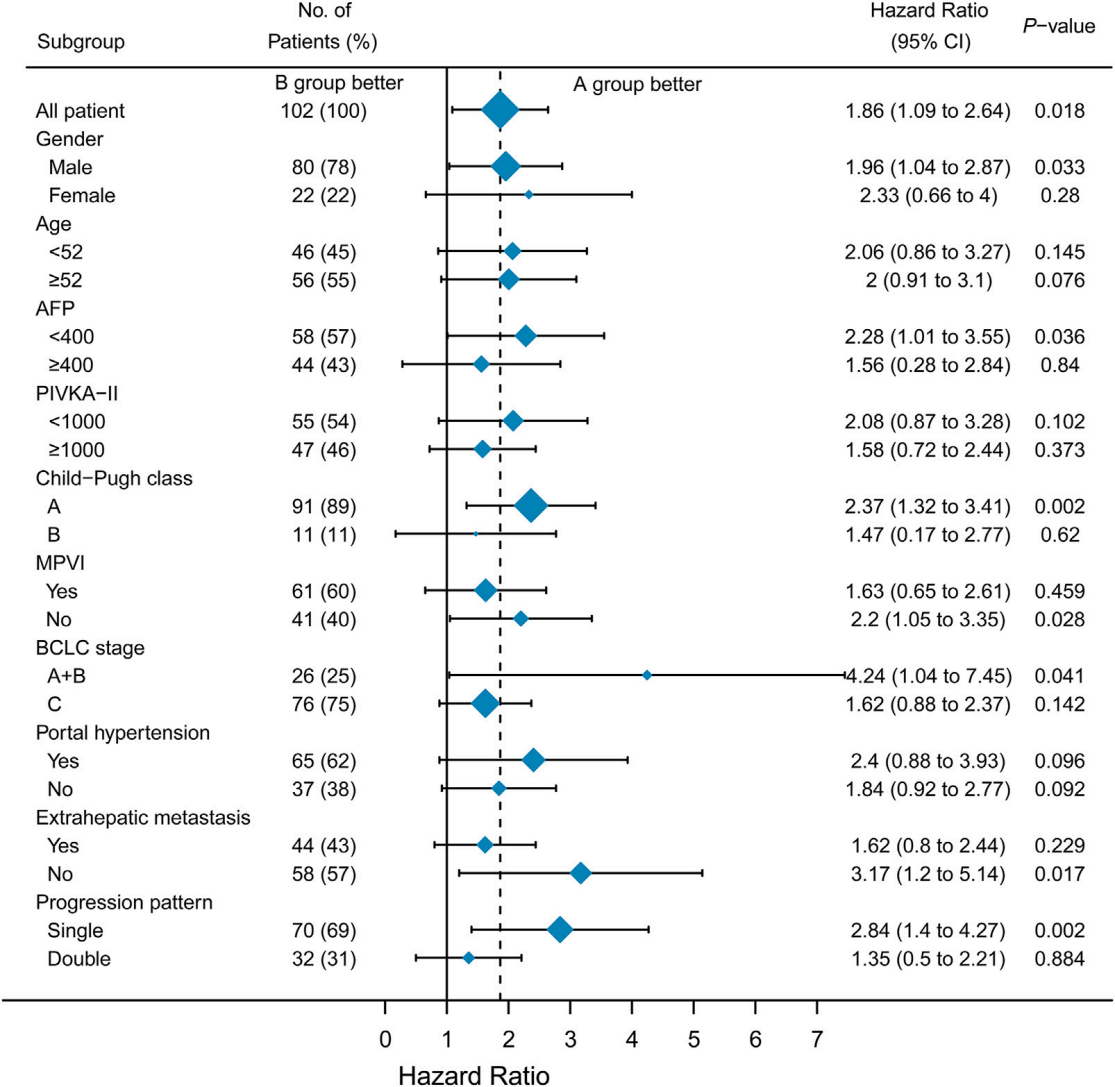
Subgroup analysis of second progression-free survival. MPVI, macroscopic portal vein invasion.

**FIGURE 4:**
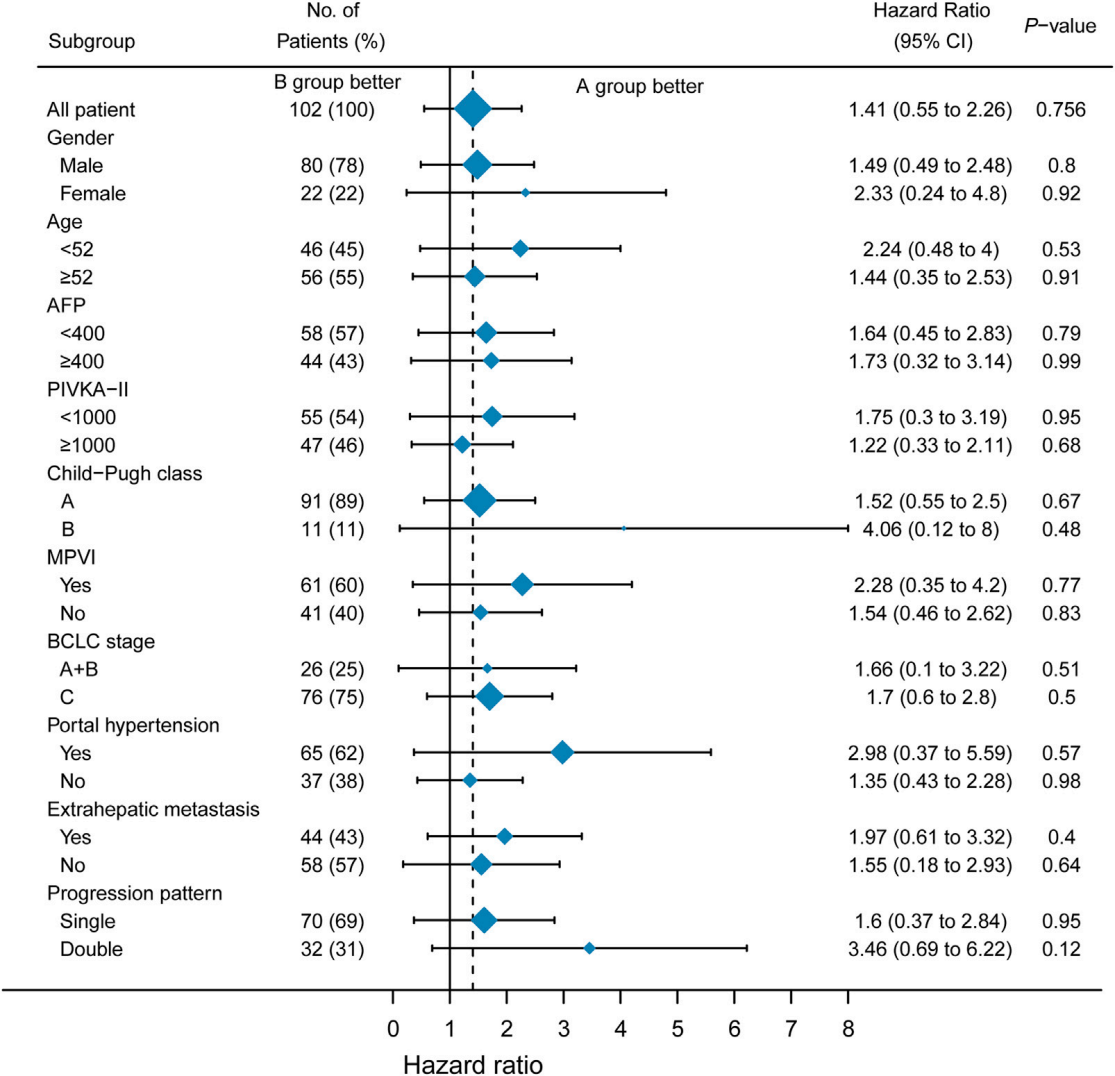
Subgroup analysis of second overall survival. MPVI, macroscopic portal vein invasion.

A subgroup analysis was performed to identify the subset of patients who could benefit from a single drug-switching strategy. Interestingly, single drug switching strategy extended the SPFS of HCC patients with AFP<400 ng/ml (HR = 1.89, 95%CI: 1.01–3.55, *p* = 0.0365), Child-Pugh class A (HR = 2.12, 95%CI: 1.32–3.41, *p* = 0.0018), absence of macroscopic portal vein invasion (HR = 1.88, 95%CI: 1.05–3.35, *p* = 0.0275), BCLC stage A or B (HR = 2.78, 95%CI: 1.04–7.45, *p* = 0.0414), absence of extrahepatic metastasis (HR = 2.48, 95%CI: 1.20–5.14, *p* = 0.0166), and single progression pattern (HR = 2.45, 95%CI:1.40–4.27, *p* = 0.0019). However, SPFS was not extended in patients with macroscopic portal vein invasion (Figure 3). No significant difference in OS was observed among the different subgroups (Figure 4). Collectively, the mode of single drug switching could extend SPFS in patients, especially in those without BCLC stage A or B.

### Lenvatinib increased the second progression-free survival in the single drug-switching group

**FIGURE 5:**
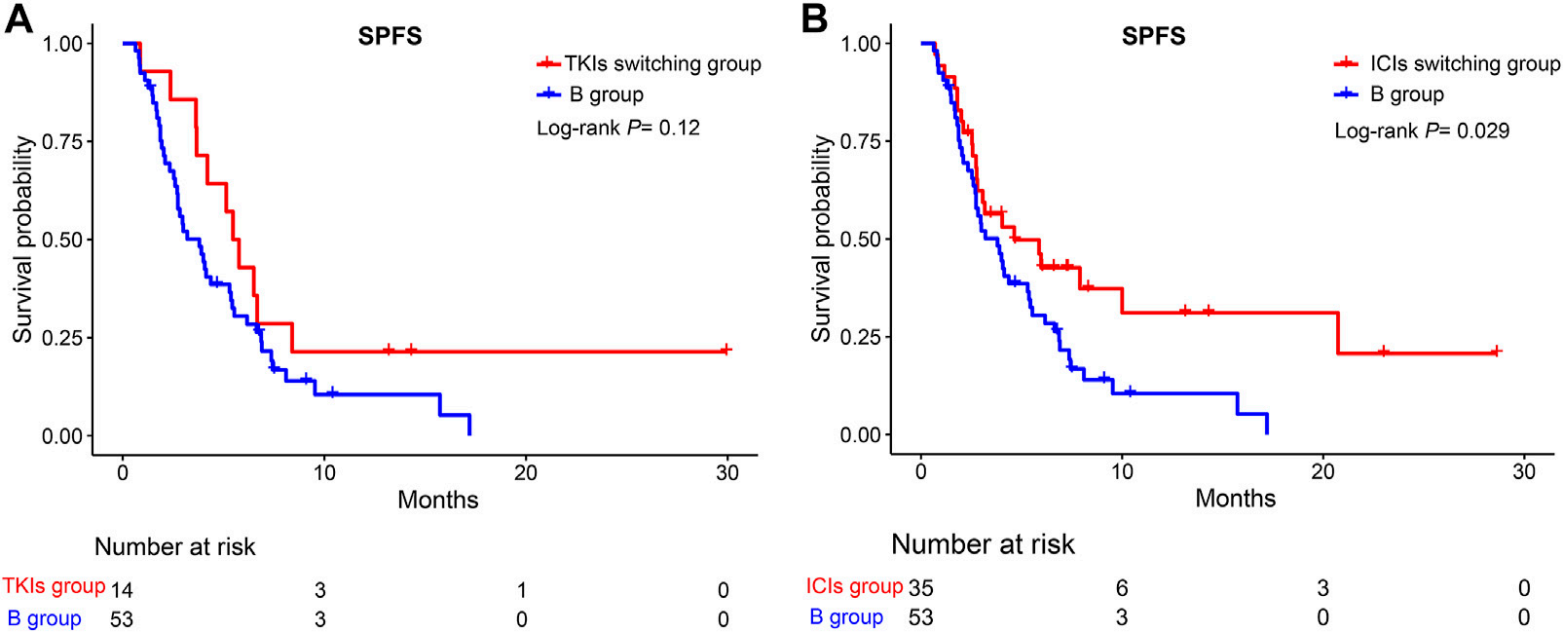
Kaplan-Meier curves for SPFS of patients in the TKIs switching group, ICIs switching group, and the B group. **(A)** TKIs switching group vs. B group; **(B)** ICIs switching group vs. B group. SPFS, second progression-free survival; TKIs, tyrosine kinase inhibitors; ICIs, immune checkpoint inhibitors.

**FIGURE 6:**
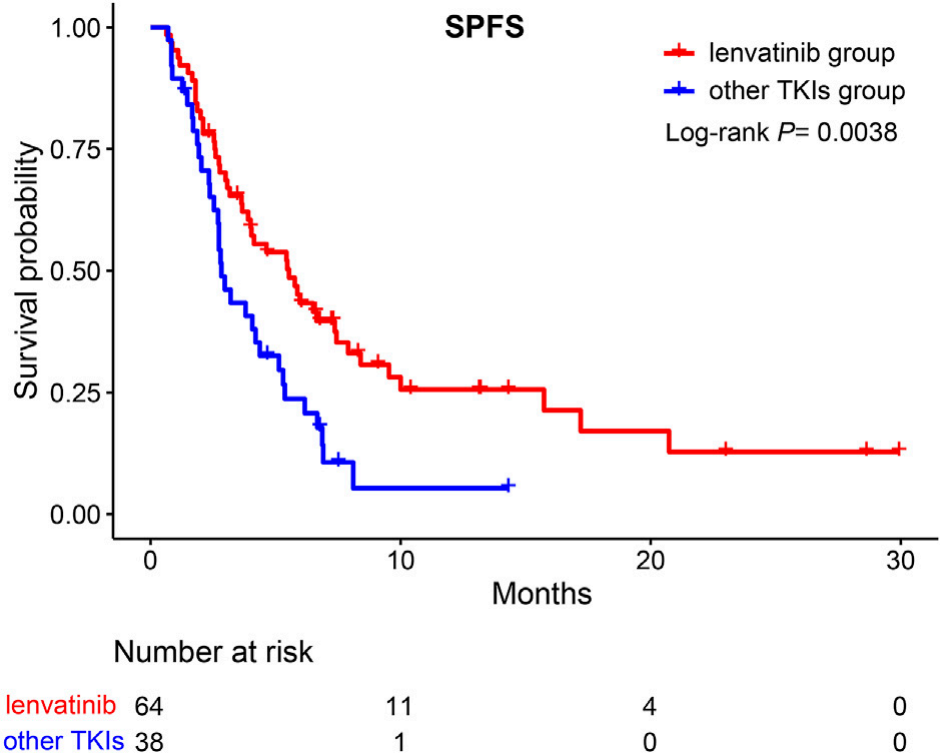
Kaplan-Meier curves for SPFS of patients moving to second-line therapy. SPFS, second progression-free survival; TKIs, tyrosine kinase inhibitors.

We further divided group A into TKIs switching and ICIs switching groups. No significant difference was observed between the TKIs switching and B groups (HR = 0.63, 95%CI; 0.35–1.13, *p* >0.05) (Figure 5A). However, compared to group B, the ICIs switching sub-group could significantly extend the SPFS (HR = 0.58, 95%CI: 0.36–0.95, *p* = 0.029) (Figure 5B). The majority of uHCC patients in the ICIs switching group retained lenvatinib. Based on these results, we hypothesized that lenvatinib could be an important factor affecting the treatment efficacy. Thus, the effectiveness of lenvatinib treatment with other TKI treatments as second-line therapies was compared. As shown in Figure 6, lenvatinib treatment accounted for better SPFS than other TKI treatments (5.53 vs. 2.83 months, *p* = 0.0038).

### Lenvatinib in the comprehensive treatment for unresectable hepatocellular carcinoma

**FIGURE 7:**
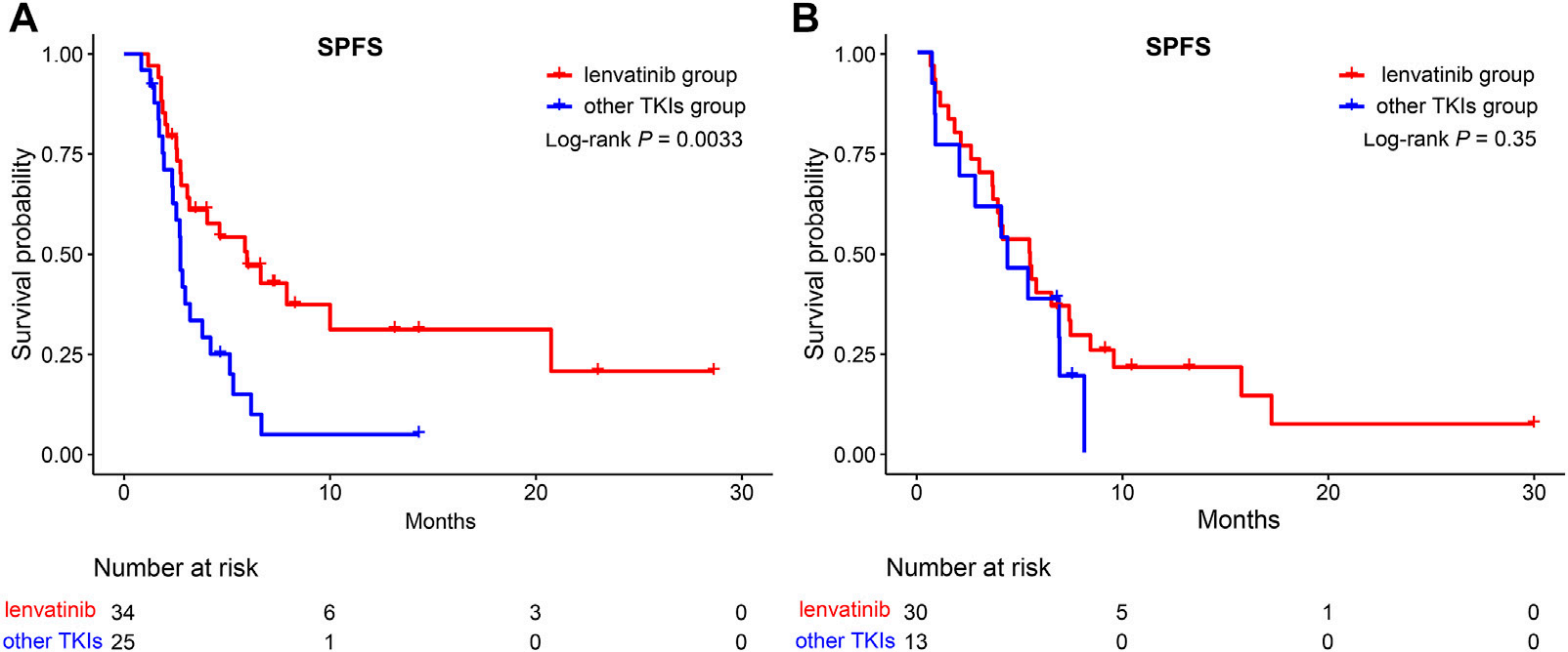
Kaplan-Meier curves for SPFS of patients in the lenvatinib group and the other TKIs group. **(A)** A subgroup of patients after lenvatinib as first-line therapy; **(B)** A subgroup of patients after other TKIs as first-line therapy. SPFS, second progression-free survival; TKIs, tyrosine kinase inhibitors.

In addition, the efficacy of lenvatinib as a first-line sequential treatment was investigated. As shown in Figure 7, patients who received lenvatinib as first-line therapy, compared to other TKIs treatments, could still benefit from retaining lenvatinib as the second-line treatment (5.97 vs. 2.73 months, *p* = 0.0033). However, for those patients receiving other TKIs treatment as a first-line treatment, no survival benefit was reported between lenvatinib and other TKIs treatments in the second-line treatment (5.43 vs. 4.36 months, *p* >0.05).

### Safety analysis

As shown in Table 5, no AE-associated deaths were observed during the follow-up. The most common AEs were increased AST, followed by increased ALT, and pain in both groups. Seven (14.3%) and eleven (20.8%) patients in groups A and B experienced at least one grade 3/4 AE. Grade 3 AEs are severe, serious, or medically significant but not immediately life-threatening, requiring hospitalization or prolonged hospitalization and partial loss of self-care. Grade 4 AEs are life-threatening, which may lead to fatal consequences, and urgent intervention is required. The AEs in Groups A and B were manageable.

**TABLE 5:** Treatment-related adverse events.

Adverse events	Any grade			Grade 3/4		
	A group (*n* = 49)	B group (*n* = 53)	*p*-value	A group (*n* = 49)	B group (*n* = 53)	*p*-value
Treatment-related AEs, n (%)						
Rash	2 (4)	6 (11.3)	0.3221	0 (0)	1 (1.9)	1
Pruritus	2 (4)	2 (3.8)	1	0 (0)	1 (1.9)	1
Pain	11 (22)	14 (26.4)	0.6541	2 (4.1)	4 (0)	0.4574
Fever	2 (4)	4 (7.5)	0.7474	0 (0)	0 (0)	1
Diarrhea	6 (14)	4 (7.5)	0.6427	0 (0)	0 (0)	1
Fatigue	4 (8)	3 (5.7)	0.9143	0 (0)	0 (0)	1
Nausea	2 (4)	3 (5.7)	0.7122	0 (0)	0 (0)	1
Decreased appetite	5 (10)	6 (11.3)	0.8559	0 (0)	0 (0)	1
Cough	6 (12)	4 (7.5)	0.6427	2 (4.1)	1 (1.9)	0.9450
Edema periphera	3 (6)	1 (1.8)	0.5548	1 (2.0)	0 (0)	0.4804
Hypothyroidism	2 (4)	3 (5.7)	1	0 (0)	0 (0)	1
Hyperthyroidism	0 (0)	1 (1.9)	1	0 (0)	0 (0)	1
Laboratory-related AEs, n (%)						
White blood cell count decreased	3 (6)	3 (5.7)	0.9211	0 (0)	0 (0)	1
Hemoglobin decreased	4 (8)	8 (15.1)	0.4366	0 (0)	2 (3.8)	0.4958
Platelet count decreased	7 (14.3)	6 (11.3)	0.6537	1 (2.0)	2 (3.8)	0.6048
Neutropenia	1 (2)	2 (3.8)	0.6048	1 (2.0)	0 (0)	0.9553
Alanine aminotransferase increased	15 (30.6)	21 (39.6)	0.3414	1 (2.0)	0 (0)	0.4804
Aspertate aminotransferase increased	18 (36.7)	28 (52.8)	0.1026	1 (2.0)	3 (5.7)	0.6669
Total bilirubin increased	9 (18.4)	8 (15.1)	0.6577	2 (4.1)	0 (0)	0.2283
Albumin decreased	9 (18.4)	10 (18.9)	0.9483	0 (0)	0 (0)	1
Creatinine increased	1 (2)	2 (3.8)	0.6048	0 (0)	0 (0)	1

Abbreviation: AEs, adverse events.

### Prognostic factors for second progression-free survival and overall survival

The results of univariate Cox regression analysis indicated that AFP≥400 (HR = 1.797, *p* = 0.0116), BCLC stage C (HR = 1.959, *p* = 0.0173), Child-Pugh class B (HR = 2.649, *p* = 0.0049), extrahepatic metastasis (HR = 1.769, *p* = 0.0165), PIVKA-II≥1,000 (HR = 1.874, *p* = 0.0036), progression pattern (HR = 1.735, *p* = 0.007), and switching to two systemic therapy drugs after tumor progression (HR = 1.722, *p* = 0.0192) were potential prognostic biomarkers of SPFS. The potentially predictive biomarkers were introduced into multivariate Cox regression analysis which confirmed that Child-Pugh class B (HR = 4.060, *p* <0.001) and switching to two systemic therapy drugs after tumor progression (HR = 4.060, *p* = 0.0123) were independent prognostic factors for SPFS (Table 6). In addition, extrahepatic metastasis (HR = 2.212, *p* = 0.055), PIVKA-II≥1,000 (HR = 2.603, *P*= 0.0119), and progression pattern (HR = 2.684, *p* <0.001) were potential prognostic biomarkers for OS. Further analysis indicated that PIVKA-II≥1,000 (HR = 2.651, *P*= 0.0118) was an adverse prognostic factor for OS (Table 7).

**TABLE 6:** Univariable and multivariable Cox regression analyses for second progression-free survival.

Characteristic	Univariate analysis		Multivariable analysis	
	HR (95% CI)	*P*	HR (95% CI)	*P*
Age (</≥52)	0.969 (0.620–1.515)	0.89		
Gender, (female/male)	1.002 (0.597–1.680)	0.995		
AFP (ng/ml), (</≥400)	1.797 (1.140–2.833)	0.0116	1.462 (0.817–2.617)	0.201
PIVKA-II, (mAU/ml), (</≥1,000)	1.957 (1.246–3.074)	0.0036	1.325 (0.753–2.330)	0.329
Child-Pugh (A/B)	2.649 (1.344–5.224)	0.0049	4.052 (1.806–9.094)	0.0007
BCLC (A + B/C)	1.959 (1.126–3.408)	0.0173	0.885 (0.395–1.979)	0.765
Extrahepatic metastasis (no/yes)	1.757 (1.108–2.785)	0.0165	1.892 (0.926–3.865)	0.0802
Macroscopic portal vein invasion (no/yes)	1.229 (0.783–1.929)	0.371		
Portal hypertension (no/yes)	1.126 (0.706–1.793)	0.619		
Progressive-pattern (single/both)	1.897 (1.191–3.019)	0.007	1.644 (0.988–2.736)	0.056
Drug switching group (A group/B group)	1.722 (1.093–2.712)	0.0192	1.844 (1.142–2.978)	0.0123

**TABLE 7:** Univariable and multivariable Cox regression analyses for overall survival.

Characteristic	Univariate analysis		Multivariable analysis	
	HR (95% CI)	*P*	HR (95% CI)	*P*
Age (</≥52)	0.98 (0.476–2.020)	0.957		
Gender, (female/male)	0.938 (0.402–2.188)	0.883		
AFP (ng/ml), (</≥400)	1.029 (0.493–2.146)	0.94		
PIVKA-II, (mAU/ml), (</≥1,000)	2.603 (1.235–5.491)	0.0119	2.651 (1.242–5.662)	0.0118
Child-Pugh (A/B)	1.910 (0.663–5.500)	0.23		
BCLC (A + B/C)	1.851 (0.707–4.845)	0.21		
Extrahepatic metastasis (no/yes)	2.212 (0.984–4.971)	0.055	1.889 (0.786–4.536)	0.155
Macroscopic portal vein invasion (no/yes)	0.852 (0.397–1.831)	0.682		
Portal hypertension (no/yes)	0.69 (0.307–1.554)	0.371		
Progressive-pattern (single/both)	2.826 (1.375–5.809)	0.005	2.072 (0.954–4.501)	0.066
Drug switching group (A group/B group)	1.121 (0.545–2.306)	0.756		

## Discussion

The treatment of uHCC is primarily based on systemic therapy. The age of patients undergoing combination treatment with TKIs and ICIs has decreased. There is abundant evidence to support that uHCC patient can benefit from a combination treatment of TKIs and ICIs (Cheng et al., 2020b). However, for HCC patients who progress on first-line combination treatment, many treatment options are available for subsequent therapies. Moreover, there is still a lack of generally accepted guidelines to guide second-line therapy after the progression of first-line combination treatment. There are two strategies for switching drugs in clinical practice: single drug switching (group A) and double drug switching (group B). This retrospective study aimed to evaluate and compare the effectiveness of two strategies of drug switching for patients with HCC who had failed combination treatment with TKIs and ICIs based on real-world data from clinical practice.

In our study, 102 patients with HCC were divided into groups A (*n* = 49) and B (*n* = 53). We observed a higher ORR (16.3%) and DCR (61.2%) in the group A. Further survival analysis indicated a significant difference in SPFS between groups A and B. Surprisingly, the median SPFS of group A was longer than that of group B (5.47 vs. 3.8 months, *p* = 0.0176). These data suggest that the median SPFS in our study was significantly extended compared to that of a previous study where the sequence ramucirumab for uHCC after TKI treatment (Amioka et al., 2021).

However, we observed no differences in the OS between groups A and B. The reason for this may be as follows. First, the follow-up time for SPFS was shorter and the sample size for SPFS was smaller than for OS. Our study’s sample size and follow-up time might not be sufficient for OS calculation. Second, OS might be affected by subsequent treatment and does not directly reflect the true efficacy of switching therapy. After switching therapy, patients with uHCC may receive other subsequent treatments, such as interventional therapy and radiotherapy. We did not observe a significant difference in OS between groups A and B.

We further analyzed which subgroup of patients could benefit from a single drug switch and double drug switch. In the subgroup analysis, we found that HCC patients with AFP<400, Child-Pugh class A, without macroscopic portal vein invasion, BCLC stage A or B, without extrahepatic metastasis, and a single progression pattern could benefit from the single drug switching strategy. In our study, Child-Pugh class A was associated with a better prognosis. A previous study demonstrated that uHCC patients with Child-Pugh class A could receive a sufficient relative dose intensity of lenvatinib, which sequentially affected the objective response (Sasaki et al., 2019). AFP level is used for the diagnosis of HCC. Previous studies have shown that there is a close relationship between AFP levels and response to comprehensive treatment (Chau et al., 2018). Consistent with a previous study, AFP < 400 ng/ml could predict the response to a single drug-switching strategy (Regmi et al., 2021). BCLC staging is a generally acknowledged system for the treatment of HCC (Reig et al., 2022). As for the single progression pattern, the reason it could benefit from single drug switching may be associated with the microenvironment. The sole progression pattern indicates that one of the tumor sites may be curbed or eradicated. However, this hypothesis requires further investigation. Macroscopic portal vein invasion and extrahepatic metastasis are the parameters of BCLC stage C. Mei et al. (2021) demonstrated that HCC patients with main portal vein tumor thrombus or extrahepatic metastasis could not benefit most from hepatic artery infusion chemotherapy plus lenvatinib combination therapy. In our study, these results indicate that the single-drug switching strategy might be suitable for patients with BCLC stage A or B. BCLC stage C indicates a more malignant tumor. As a result, compared with uHCC patients with stage A or B disease, patients with macroscopic portal vein invasion or extrahepatic metastasis seemed to be more inclined to progress, leading to a worse survival prognosis. Collectively, the mode of single drug switching could extend SPFS in patients, especially in those with BCLC stage A or B.

In our study, patients could benefit from single-drug switching rather than double-drug switching. To explain the reasons for this result, we further divided group A into TKIs switching and ICIs switching groups. Surprisingly, compared with group B, the ICIs switching group could significantly extend the SPFS. However, no significant difference was reported between the TKIs switching and the B group. Both uHCC patients in the ICIs switching group and B group switched ICIs after tumor progression. Why could the former group extend the SPFS? We found that the majority of uHCC patients in the ICIs switching group retained lenvatinib. Moreover, for second-line therapy, lenvatinib treatment accounted for a better SPFS than other TKI treatments (5.53 vs. 2.83 months, *p* = 0.0038). This result further confirms our hypothesis. The REFLECT clinical trial indicated that the overall survival time of the lenvatinib group was not inferior to the sorafenib group (Kudo et al., 2018). Further studies indicated that, compared with sorafenib, lenvatinib exhibited stronger inhibitory activity targeting the fibroblast growth factor receptor (Tohyama et al., 2014). Shi et al. (2021) found that lenvatinib may be a suitable second-line treatment for uHCC patients who progressed on sorafenib by regulating FGFR4-ERK signaling. Apatinib is a small-molecule tyrosine kinase inhibitor that selectively inhibits the activity of VEGFR-2 (Tian et al., 2011). Moreover, a previous study indicated that lenvatinib had immunomodulatory activity, which contributed to the antitumor effect of lenvatinib and enhanced the synergistic effect with the anti-PD-1 antibody (Kimura et al., 2018). Moreover, Chen et al. demonstrated that lenvatinib could reduce the expression of PD-L1 in HCC and regulate T-cell differentiation by blocking FGFR4 to improve anti-PD-1 efficacy (Yi et al., 2021). Collectively, retaining lenvatinib accounted for the survival benefits of single-drug switching, especially in SPFS. However, lenvatinib led to better SPFS, but did not translate into OS benefits. The use of longer SPFS with lenvatinib to enable patients to obtain longer OS benefits still needs to be explored by oncologists.

Further analysis indicated that for those patients who selected lenvatinib as the first-line treatment, compared to other TKIs treatment, they could still benefit from retaining lenvatinib as the second-line treatment (5.97 vs. 2.73 months, *p* = 0.0033). However, for patients who selected other TKIs as the first-line treatment, no survival benefit was reported between lenvatinib and other TKIs treatments. Chen et al. retrospectively analyzed 26 cases of advanced uHCC from October 2018 to October 2019 in the real world in China and found that lenvatinib combined with the PD-1 antibody was expected to further improve the prognosis of patients who progressed on lenvatinib (Chen et al., 2020). Thus, lenvatinib should be used for the comprehensive treatment of uHCC. However, high-quality randomized controlled studies are required to validate this conclusion.

In the prognostic factor analysis, the Child-Pugh class and drug-switching strategy were identified as independent prognostic factors for SPFS. The Child-Pugh class is an evaluation system for liver reserve function, including five parameters (Kok and Abraldes, 2019). In our study, Child-Pugh class A could predict better SPFS, and HCC patients with Child-Pugh class A could obtain a longer SPFS benefit from the single drug switching strategy. The reason for this might be that HCC patients with Child-Pugh class A could better tolerate the combination therapy’s toxicity. Moreover, a PIVKA-II>1,000 was regarded as an adverse prognostic factor for OS. Another prognostic factor is the drug-switching strategy. Based on the results of the comparison of the two drug-switching strategies in clinical practice, we found that single-drug switching could extend the SPFS. PIVKA-II is produced because of the incomplete carboxylation of amino acid residues (Liebman et al., 1984). What is clear is that PIVKA-II is not only a diagnostic predictor but also a prognostic predictor of liver cancer (Yang et al., 2021). PIVKA-II exhibited stronger mitogenic capacity and migratory activity during angiogenesis in HCC patients (Bertino et al., 2010).

As for safety, consistent with a previous study, toxicities were manageable with no unexpected safety signals (Mo et al., 2021). No AE-associated death was observed during follow-up, and the most common AEs were damage to liver function. Dose adjustments of TKIs and ICIs accounted for safety in the present study. In our study, the percentages of interruption and dose reduction in groups A and B were 30% and 35%, respectively. Half of the routine dosage or weekends-off administration of lenvatinib (Iwamoto et al., 2020) was the primary method of dose adjustment.

We acknowledge the potential limitations of this study. First, a selection bias was unavoidable because this was a retrospective study. Liver function was worse in group A than in group B, and it was positively correlated with survival rate. However, the survival analysis indicated that the treatment response and SPFS of group A were better than those of group B. The potential selection bias worked unfavorably against the single-drug switching strategy, leading to an opposite result. Secondly, one hundred and two patients with uHCC were included in our study. The sample size was small, and the observation period was short. All included patients were Asian, and their data were obtained from a single Chinese institute. A single drug-switching strategy might be beneficial only to the Asian population. A great amount of evidence has demonstrated that the carcinogenic factors of patients with HCC in Asia and the West are different, which limits the ability to draw general conclusions from the results (Marengo et al., 2016). Collectively, our conclusion requires further confirmation by a large international multicenter clinical study in the future. Third, confounding factors are one of the limitations. We defined drug-switching strategies for second-line therapy after combination treatment with TKIs and ICIs, but the optional treatment for HCC patients lacks clear guidelines. Subsequent treatments after first-line treatment were not chosen in a randomized manner. Thus, the therapeutic molecules used in the second line might vary between groups A and B, which influenced the uniformity of the treatment procedure. Such division of patients into different groups may bring about a certain degree of heterogeneity; thus, this was a preliminary study on a drug-switching strategy for second-line therapy after combination treatment with tyrosine kinase inhibitors and immune checkpoint inhibitors for unresectable hepatocellular carcinoma. The findings of this study should be further validated using higher-level randomized controlled trials. Finally, lenvatinib was the main TKIs used in combination with ICIs in our study. Thus, the value of other TKIs, such as sorafenib and regorafenib, in combination treatment should be further investigated.

## Conclusion

After combination treatment with TKIs and ICIs failure, single-drug switching significantly prolonged the median SPFS in uHCC patients, and retaining lenvatinib accounted for the survival benefit brought by single-drug switching.

## Data Availability

The original contributions presented in the study are included in the article/Supplementary Material, further inquiries can be directed to the corresponding authors.

## Abbreviations

AEs, Adverse events; AFP, α-fetoprotein; ALP, Alkaline phosphatase; ALT, Alanine aminotransferase; AST, Aspartate aminotransferase; BCLC, Barcelona Clinic Liver Cancer stage; CR, Complete response; DCR, Disease control rate; HCC, Hepatocellular carcinoma; HR, Hazards ratio; ICIs, Immune checkpoint inhibitors; OS, Overall survival; ORR, Objective response rate; PD, Progressive disease; PFS, Progression-free survival; PIVKA-II, Protein induced by vitamin K absence or antagonist-II; PR, Partial response; SD, Stable disease; SPFS, Second progression-free survival; TB, Total bilirubin; TKIs, Tyrosine kinase inhibitors; uHCC, Unresectable hepatocellular carcinoma.
