# Supplementary material for: A preliminary study on drug switching strategy for second-line therapy after combination treatment of tyrosine kinase inhibitors and immune checkpoint inhibitors for unresectable hepatocellular carcinoma
Source: Front Pharmacol. 2022 Sep 30;13:998534. doi: 10.3389/fphar.2022.998534 (PMC9561133; doi:10.3389/fphar.2022.998534)
Supplement: Supplementary file 1 [file Table1.docx]

Supplement Table1. Category and dosage of tyrosine kinase inhibitors and PD-1 inhibitors used in the initial treatment.

| Category | Dose  (mg) | Number of patients |
| --- | --- | --- |
| Tyrosine kinase inhibitors |  |  |
| Lenvatinib | 8 | 59 |
| Sorafenib | 400 | 20 |
| Regorafenib | 40 | 6 |
| Apatinib | 250 | 17 |
| Immune checkpoint inhibitors |  |  |
| Nivolumab | 100 | 7 |
| Keytruda | 200 | 14 |
| Toripalimab | 240 | 49 |
| Sintilimab | 200 | 24 |
| Camrelizumab | 200 | 5 |
| Tislelizumab | 200 | 1 |
| Durvalumab | 620 | 2 |
